# Fecal microbiome associated with egg production efficiency in laying hens

**DOI:** 10.5713/ab.25.0256

**Published:** 2025-10-22

**Authors:** Jr-Wei Chen, Sheng-Yao Wang, Wen-Yuan Yang

**Affiliations:** 1 Department of Animal Science and Technology, National Taiwan University, Taipei City, Taiwan; 2 Department of Animal Industry, Ministry of Agriculture, Executive Yuan, Taipei City, Taiwan; 3 Department of Veterinary Medicine, School of Veterinary Medicine, National Taiwan University, Taipei City, Taiwan

**Keywords:** Egg Production, Fecal Microbiome, Firmicutes-to-Bacteroidetes Ratio, Full-length 16S rRNA Gene Sequencing, *Lactobacillus*

## Abstract

**Objective:**

This study aimed to characterize the fecal microbiome of highly productive laying hens to identify microbial signatures linked with enhanced egg production performance.

**Methods:**

Six commercial layer farms were enrolled in the study. Farms with an average monthly egg production rate (amEPR) above 80% over six consecutive months were classified as the high-production group (D group; n = 3), while those with amEPR below 60% were designated as the low-production group (P group; n = 3). All hens were raised in open-type housing systems without a history of forced molting. From each farm, 36 fecal samples were randomly collected and subjected to full-length 16S ribosomal RNA gene sequencing to characterize the microbiome.

**Results:**

The D group showed significantly higher Firmicutes-to-Bacteroidetes ratios (p< 0.001) and lower species richness (Menhinick index, p<0.05) compared with the P group. Multivariate analyses (Adonis, ANOSIM, and MRPP; all p<0.001) revealed distinct microbial community structures between the groups. Taxonomic profiling indicated that the D group harbored higher relative abundances of *Enterococcus cecorum* and *Lactobacillus kitasatonis* (both p<0.05). In contrast, the P group had elevated levels of *Bacteroides eggerthii* and *Bacteroides coprosuis* (p<0.001 and p<0.05, respectively). Principal component analysis and linear discriminant analysis effect size further identified *Enterococcus cecorum* and *Lactobacillus kitasatonis* as biomarkers associated with superior egg production. In Hy-Line hens, *Lactobacillus gallinarum* and *Lactobacillus salivarius* were also identified as biomarkers linked to high productivity.

**Conclusion:**

The fecal microbiome of highly productive laying hens is characterized by an enrichment of *Enterococcus cecorum* and lactic acid bacteria, particularly *Lactobacillus kitasatonis*, *Lactobacillus gallinarum*, and *Lactobacillus salivarius*. Their targeted supplementation may represent a promising probiotic strategy to improve egg production efficiency in commercial laying hens.

## INTRODUCTION

Chicken eggs are an essential and affordable source of high-quality animal protein and nutrients for human consumption. In 2018, global egg production reached approximately 82.8 million tons, and the demand for this vital source of animal protein continues to rise [1]. Ensuring stable and efficient egg production is, therefore, critical for meeting the nutritional needs of a growing population while simultaneously minimizing the environmental impact of poultry farming [2]. However, the current egg production systems in Asia, particularly the open-type cage housing systems, present significant challenges for commercial egg laying. Declining egg production and disruptions in laying sequences are common concerns threatening egg productivity in this system. Addressing strategies to stabilize egg output is thus imperative to support the sustainability of poultry production and the availability of this indispensable source of protein.

Recent studies have emphasized the significant role of the gut microbiota in enhancing reproductive performance in laying hens. The gut microbiota develops rapidly during the first three days after hatching, with most microorganisms in the mature microbiota established by day seven [3]. By three to six weeks of age, a relatively stable microbial community dominated by Firmicutes and Bacteroidetes is established [4]. Disruptions to this balance, known as dysbiosis, can lead to metabolic disorders, inflammatory diseases, and diminished productivity [5]. Several studies further demonstrated a significant correlation between the composition of the intestinal microbiome and the health and productivity of hens [6]. These findings underscore the potential of modulating the gut microbiota to enhance egg production efficiency in the poultry industry. Furthermore, chickens with diverse and beneficial gut microbiota exhibit more excellent resistance to intestinal pathogens, supporting the importance of maintaining microbial balance [5,7].

DNA sequencing methods represent a cornerstone in microbiome research, with approaches such as 16S ribosomal RNA (rRNA) gene sequencing and metagenomics established as powerful tools for microbial characterization [8]. Among these, the sequencing of the V3-V4 region of the 16S rRNA gene is frequently utilized due to its affordability and high-throughput capabilities, making it a practical choice for large-scale studies [9]. Despite its widespread use, short-read sequencing inherently limits taxonomic resolution at the species level. In contrast, full-length 16S rRNA sequencing offers significantly enhanced accuracy, enabling detailed species-level classification [10]. Comparative analyses revealed that while short-read sequencing provides robust correlations at higher taxonomic levels, it often fails to achieve concordance at the species level [11]. This limitation highlights the superior utility of full-length sequencing for studies requiring species-level precision. Adopting the full-length technique in microbiome research is advantageous for identifying microbial species associated with phenotypic traits of interest, further evaluated for their potential role in enhancing egg production efficiency.

The gut microbiome is well-recognized for supporting intestinal health and influences the overall performance of chickens [12]. However, the relationship between the microbiome and egg-laying performance in hens raised in open-type cage housing systems is rarely investigated. The study aims to characterize the differential community structure in high- and low-productive laying hens using full-length 16S rRNA sequencing. Fecal samples were selected for analysis because they can be obtained non-invasively and are capable of routine monitoring. Furthermore, bioinformatic approaches were applied to identify microbiome biomarkers associated with superior egg production. These findings aid in identifying predictive microbial markers for selecting highly productive hens and provide insights into potential candidates for enhancing egg production efficiency in commercial laying hens.

## MATERIALS AND METHODS

### Study population and farm selection

Six commercial layer farms were selected for fecal microbiome analysis based on the following criteria: use of open-type cage housing systems, absence of any history of forced molting, and availability of monthly egg production rate (mEPR) records from December 2020 to May 2021. Farms with an average mEPR (amEPR) above 80% over the six-month period were enrolled as the high-production group (D group), while those with an amEPR below 60% were categorized as the low-production group (P group). The D group included farms D1, D2, and D3, all of which raised Hy-Line White laying hens. The P group comprised farms P1, P2, and P3, which housed either Hendrix or Hy-Line White laying hens. All farms used commercial layer feed, and no antibiotics were administered through feed, water, or other routes during the laying period. Additional details regarding flock size, rearing conditions, average egg production rates, and hen ages are provided in Table 1.

### Sample collection

A two-tailed power analysis indicated that a minimum of 34 samples was required to detect microbial differences between two farms, based on an effect size of 0.7, a significance level of p<0.05, and a statistical power of 0.8. In this study, 36 fecal samples were randomly and evenly collected from each farm, with samples distributed across different areas within the poultry houses to ensure spatial representativeness. Only freshly excreted feces with a paste-like consistency and light brown color were collected, while the uric acid-rich white portions were excluded to ensure that the bacterial composition accurately reflected the intestinal microbiota. Fecal material was aseptically transferred into sterile tubes using sterilized spoons. All samples were immediately placed on dry ice after collection and transported to the laboratory for further processing.

### Nucleic acid extraction and 16S rRNA full-length sequencing

For each farm, 36 fecal samples were pooled into six composite samples for analysis. According to the manufacturer's protocol, the genomic DNA was extracted from 250 mg of a composite sample using the CatchGene Stool DNA Kit (Qiagen). DNA concentration and quality were assessed using the Qubit 4.0 fluorometer (Thermo Fisher Scientific) and gel electrophoresis. Subsequently, the full-length 16S rRNA gene (V1-V9 regions) was amplified with the barcoded universal primer set (forward: 5′ GCATC/barcode/AGRGTTYGA-TYMTG GCTCAG 3′, reverse: 5′ GCATC/barcode/RGYTACCTTGT TACGACTT 3′). Amplification was performed with KAPA HiFi HotStart ReadyMix PCR kit (Roche) under the following conditions: initial denaturation at 95°C for 3 minutes, 25 cycles of denaturation at 95°C for 30 seconds, annealing at 57°C for 30 seconds, and extension at 72°C for 60 seconds. A final extension at 72°C for 5 minutes was added. The PCR amplicons (~1.5 kb) were purified using AMPure PB Beads (Pacific Biosciences). Equal molar concentrations of barcoded amplicons (500–1,000 ng) were pooled for SMRTbell library preparation using the SMRTbell Prep Kit 3.0 (Pacific Biosciences). The library preparation process involved end-repair at 37°C for 30 minutes, A-tailing at 65°C for 10 minutes, adapter ligation at 20°C for 30 minutes, and nuclease treatment at 37°C for 15 minutes. Libraries were further purified with AMPure PB Beads and prepared for sequencing by annealing primers (v4) and binding polymerase using the Sequel II Binding Kit 2.1 (Pacific Biosciences). Sequencing was performed on the PacBio Sequel II system with the Sequel II Sequencing Kit 2.0 (Pacific Biosciences).

### Bioinformatics for PacBio sequence analysis

Circular consensus sequence (CCS) reads were generated using the PacBio SMRT Link software (v9), with a minimum predicted accuracy of 0.9 and at least three passes. The resulting CCS reads were processed with DADA2 (v1.14) to achieve single-nucleotide resolution amplicons. The workflow included quality filtering, dereplication, dataset-specific error modeling, amplicon sequence variant (ASV) inference, and chimera removal. Reads were then trimmed and filtered, allowing a maximum of two expected errors per read. Taxonomic classification for representative sequences was performed using the QIIME2 with feature-classifier and classify-consensus-blast algorithms, referencing the NCBI database. Multiple sequence alignment was conducted with the QIIME2 alignment MAFFT plugin to analyze sequence similarities among ASVs. The abundance of ASVs was rarefied to the minimum sequence depth for normalizing the variations in sequence depth across samples. Thereafter, alpha diversity was assessed using metrics such as Faith’s phylogenetic diversity, Menhinick’s richness, Pielou’s evenness, and the Shannon-Wiener indices. Beta diversity was analyzed using the Bray-Curtis dissimilarity metric and implemented through the “phyloseq” package in R. Analysis of similarities (Adonis, Anosim, and MRPP) was also carried out in the R package. Principal coordinate analysis (PCoA) was correspondingly performed to visualize multidimensional data based on the distance matrix. For the cluster analysis, principal component analysis (PCA) was applied to reduce the dimensions of the multiple variables, using the FactoMineR package and ggplot2 package in R software. Linear discriminant analysis effect size (LEfSe) was used to identify bacterial taxa with significant differences in abundance and assess their biological relevance. Taxa with a linear discriminant analysis (LDA) score (log10)>4 were considered significant.

### Statistical analysis

LEfSe was used to identify bacterial taxa that differed significantly in relative abundance between the high- and low-production groups. The non-parametric Kruskal-Wallis test and Wilcoxon rank-sum test were employed for group-wise comparisons. Spearman correlation analysis was performed to evaluate associations among dominant microbial species in the fecal microbiome. Laying performance data were analyzed using the Chi-square test in SAS software ver. 9.4 (SAS Institute). Statistical significance was defined as p<0.05.

## RESULTS

### Differences in egg production efficiency between groups and farms

The amEPR for each farm is summarized in Table 1. The D group exhibited a higher amEPR compared with the P group (p<0.001). Pairwise comparisons further revealed that farms D1, D2, and D3 each demonstrated greater egg production efficiency than farm P2, with all comparisons showing p<0.001.

### Microbial composition and community differences between groups

**Figure 1:**
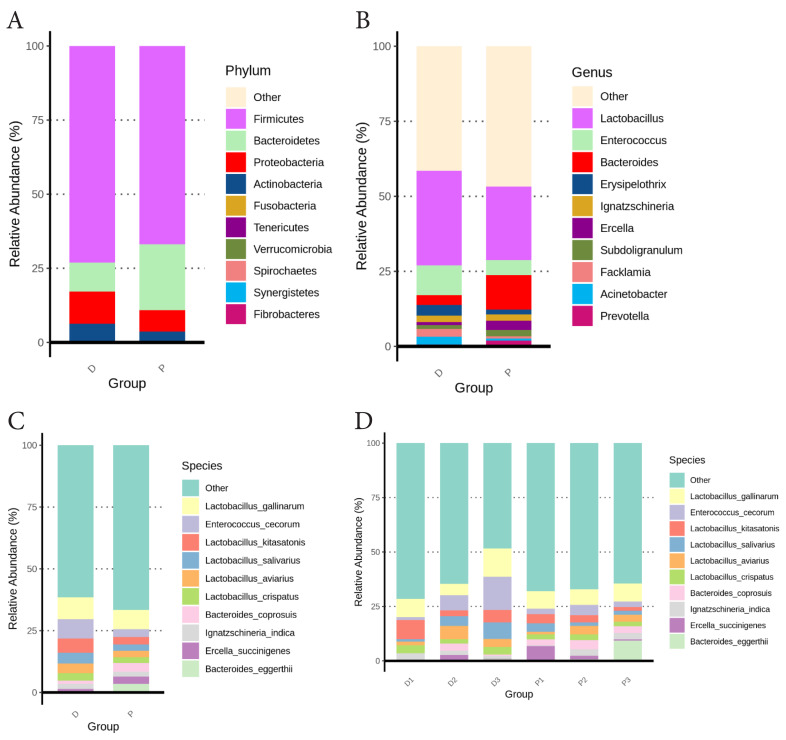
Community composition of the fecal microbiota in the D and P groups at the phylum (A), genus (B), and species (C) levels. (D) Species-level microbial composition of fecal samples from six layer farms (D1, D2, D3, P1, P2, and P3). The top ten taxa at each taxonomic rank are displayed.

Taxonomic analysis revealed the differential microbiome composition between the D and P groups. In the D group, Firmicutes accounted for the largest proportion of the microbiome (73.1%), followed by Proteobacteria (10.84%), Bacteroidetes (9.72%), Actinobacteria (6.05%), and Fusobacteria (0.15%). The P group exhibited a similar phyla composition with distinct relative abundances: Firmicutes (66.69%), Bacteroidetes (22.23%), Proteobacteria (7.19%), Actinobacteria (3.18%), and Tenericutes (0.18%; Figure 1A). Notably, the relative abundances of Bacteroidetes and Tenericutes were higher in the P group compared to the D group (p<0.01). At the genus level, the five most abundant genera in the D group were *Lactobacillus* (31.54%), *Enterococcus* (9.97%), *Erysipelothrix* (3.6%), *Bacteroides* (3.22%), and *Acinetobacter* (2.59%) (Figure 1B). The dominant genera in the P group were *Lactobacillus* (24.56%), *Bacteroides* (11.49%), *Enterococcus* (4.89%), *Ercella* (2.99%), and *Subdoligranulum* (2.14%). The D group demonstrated higher relative abundances of *Enterococcus* and *Erysipelothrix* in the pairwise comparison (p<0.05), while the P group showed higher levels of *Bacteroides* and *Ercella* (p< 0.01). At the species level, the dominant taxa in the D group included *Lactobacillus gallinarum* (8.8%), *Enterococcus cecorum* (7.86%), *Lactobacillus kitasatonis* (5.72%), *Lactobacillus salivarius* (4.44%), and *Lactobacillus aviarius* (3.81%) (Figure 1C). On the contrary, the species prevalent in the P group were *Lactobacillus gallinarum* (7.81%), *Bacteroides eggerthii* (3.39%), *Bacteroides coprosuis* (3.37%), *Enterococcus cecorum* (3.19%), and *Lactobacillus kitasatonis* (3.07%). Statistical analysis showed that the D group had higher relative abundances of *Enterococcus cecorum* and *Lactobacillus kitasatonis* (p<0.05), while the P group exhibited higher levels of *Bacteroides eggerthii* and *Bacteroides coprosuis* (p<0.05). The relative abundances of the top ten species identified in the fecal microbiome across sampled farms are presented in Figure 1D.

**Figure 2:**
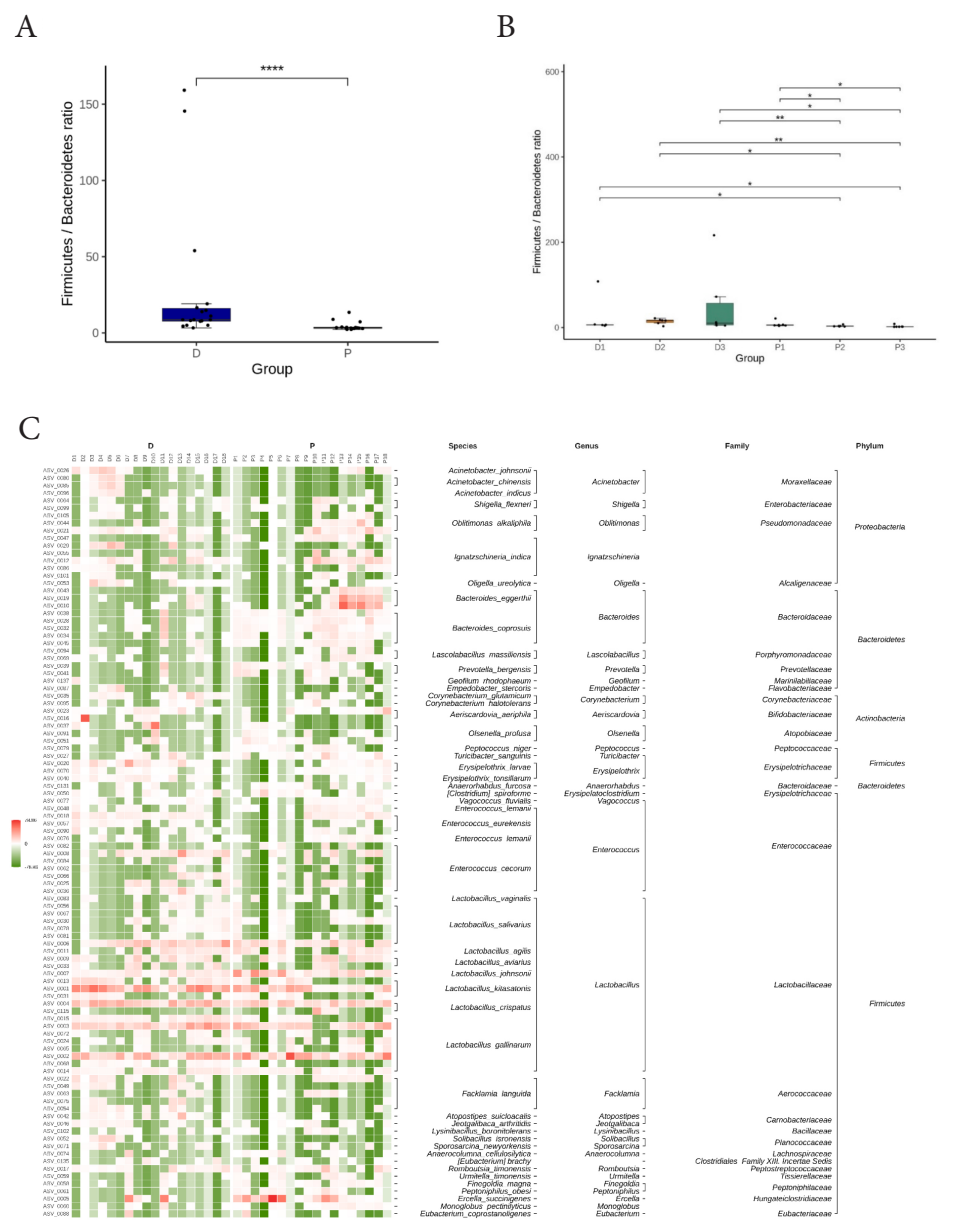
Firmicutes-to-Bacteroidetes (F/B) ratio and clustered heatmap of taxa in fecal microbiome of laying hens in farms with high production (D) and low production (P) group. (A) Comparison of F/B ratios between D and P groups using the Wilcoxon test (**** p<0.0001). (B) Comparison of F/B ratios among D1, D2, D3, P1, P2, and P3 farms using the Kruskal-Wallis test (* p<0.05; ** p<0.01). (C) The heatmap displays z-scores derived from the normalized abundance of each species across groups.

Analysis of the Firmicutes-to-Bacteroidetes (F/B) ratio showed a higher F/B ratio in the D group when compared to the P group (p<0.001; Figure 2A). Pairwise farm comparisons revealed that all farms in the D group (D1-D3) had higher F/B ratios than farms P2 and P3, respectively (Figure 2B). Heatmap-based composition and cluster analyses further highlighted clear differences in community composition between the D and P groups (Figure 2C), with *Lactobacillus kitasatonis* predominating in the D group and *Bacteroides eggerthii* dominating in the P group.

### Fecal microbiome diversity in laying hens

**Figure 3:**
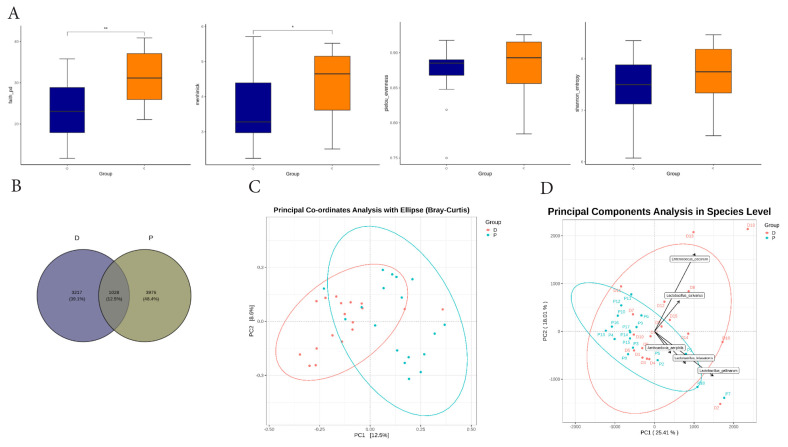
Alpha and beta diversity analyses of the fecal microbiome in laying hens. (A) Alpha diversity of the fecal microbiome shown as boxplots based on Faith’s phylogenetic diversity, Margalef richness, Pielou’s evenness, and Shannon diversity indices. Statistical significance is indicated by * p<0.05; ** p<0.01. (B) Venn diagram showing the shared and unique OTUs between the D and P groups. (C) Bray-Curtis beta diversity using principal coordinates analysis. (D) Principal component analysis plot illustrating sample distribution along the first two principal components. Arrows represent the top five microbial taxa contributing to the variance between the D and P groups. OTU, operational taxonomic unit.

Alpha diversity metrics were analyzed to assess microbial community variation within the groups. The P group exhibited higher phylogenetic diversity (Faith’s PD, p<0.01) and species richness (Menhinick, p<0.05) compared with the D group, whereas no differences were found in species evenness or diversity indices (Figure 3A). The average number of operational taxonomic units (OTUs) was 4,245 in the D group and 5,004 in the P groups. A total of 1,028 OTUs were shared between groups while 3,217 and 3,976 OTUs were unique to the D and P groups, respectively, as illustrated in the Venn diagram (Figure 3B). Compositional similarity analysis showed significant structural differences between the two groups. Adonis analysis confirmed a significant separation (p<0.001), Anosim analysis indicated that inter-group variation was greater than intra-group variation (R = 0.264, p<0.001), and MRPP analysis further supported these findings with a higher observed delta value for inter-group differences (observed delta = 0.789, p<0.001) (Table 2). The PCoA also demonstrated a distinct clustering of microbial communities, with the D and P groups occupying separate regions (Figure 3C). In addition, PCA identified key species contributing to these differences, including *Enterococcus cecorum*, *Lactobacillus gallinarum*, *Lactobacillus kitasatonis*, *Lactobacillus salivarius*, and *Aeriscadovia aeriphila* (Figure 3D).

### Taxonomic biomarkers associated with egg-lying performance

**Figure 4:**
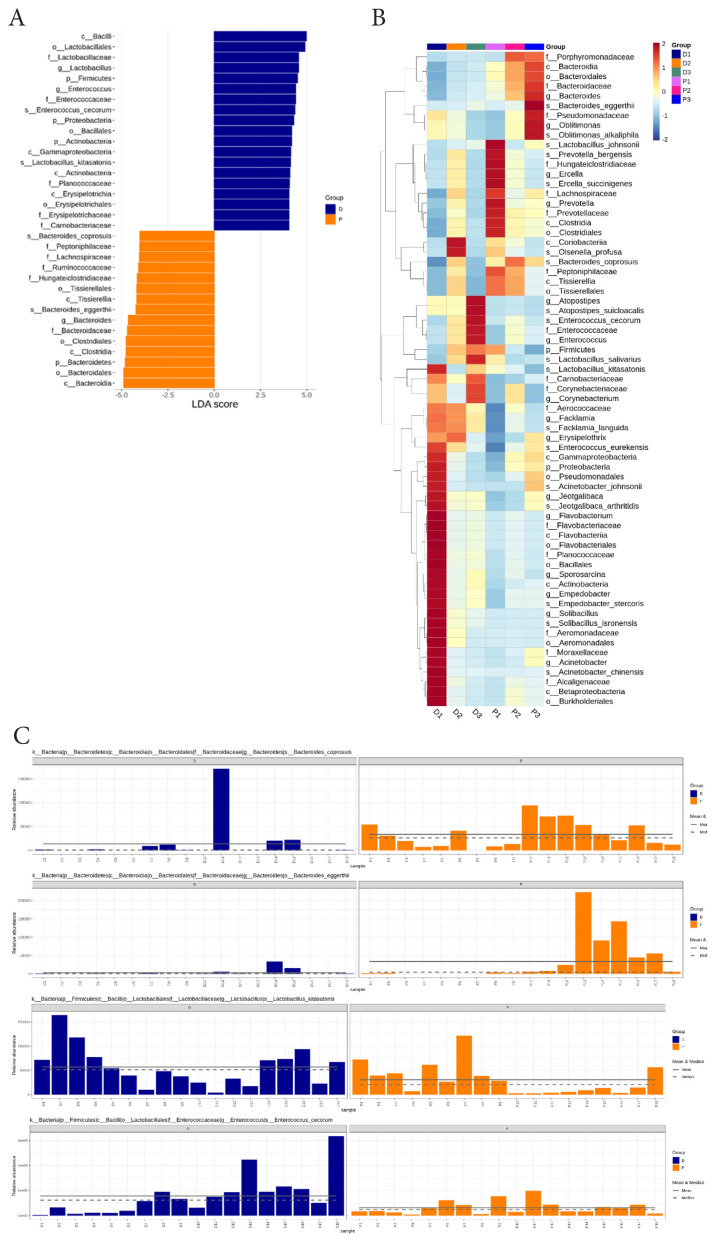
LEfSe analysis of differential microbes in the fecal microbiome between D and P groups using an LDA score threshold of 4. (A) Differential microbes (biomarkers) identified by LEfSe analysis between D and P groups. (B) Heatmap displaying differential microbes across layer farms. (C) Relative abundance of biomarkers at the species level in fecal samples. LEfSe, linear discriminant analysis effect size; LDA, linear discriminant analysis.

LEfSe analysis with an LDA threshold of 4 was conducted to identify taxa that served as differential biomarkers with the strong statistical and biological relevance. Group-level comparisons revealed *Enterococcus cecorum* and *Lactobacillus kitasatonis* as key biomarkers in the D group, whereas *Bacteroides eggerthii* and *Bacteroides coprosuis* were predominant biomarkers in the P group (Figure 4A). A heatmap illustrated the average relative abundances of these taxa across all samples (Figure 4B). Additionally, the distribution of biomarker species within individual samples from each group is presented in Figure 4C.

**Figure 5:**
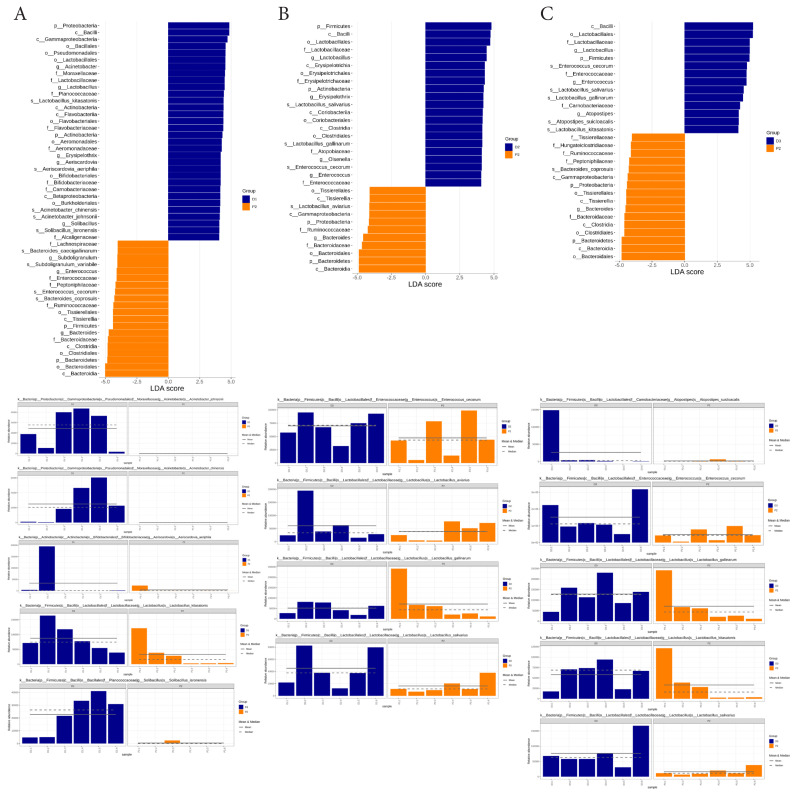
Biomarkers identified in high and low-productive Hy-Line hens using LEfSe analysis with an LDA score threshold of 4. (A) Biomarkers and their relative abundance in the comparison between D1 and P2 farms. (B) Biomarkers and their relative abundance in the comparison between D2 and P2 farms. (C) Biomarkers and their relative abundance in the comparison between D3 and P2 farms. LEfSe, linear discriminant analysis effect size; LDA, linear discriminant analysis.

Farm-level comparisons further revealed distinct biomarker profiles among Hy-Line laying hens (Figure 5). In the comparison between farms D1 and P2, *Lactobacillus kitasatonis*, *Aeriscardovia aeriphila*, *Acinetobacter chinensis*, *Acinetobacter johnsonii*, and *Solibacillus isronensis* were identified as significant biomarkers in D1. For farms D2 versus P2, *Lactobacillus salivarius*, *Lactobacillus gallinarum*, and *Enterococcus cecorum* were predominant biomarkers in D2. Similarly, the comparison between farms D3 and P2 highlighted *Enterococcus cecorum*, *Lactobacillus salivarius*, *Lactobacillus gallinarum*, *Atopostipes suicloacalis*, and *Lactobacillus kitasatonis* as significant biomarkers in D3.

### Correlation analysis of fecal microbiome biomarkers

**Figure 6:**
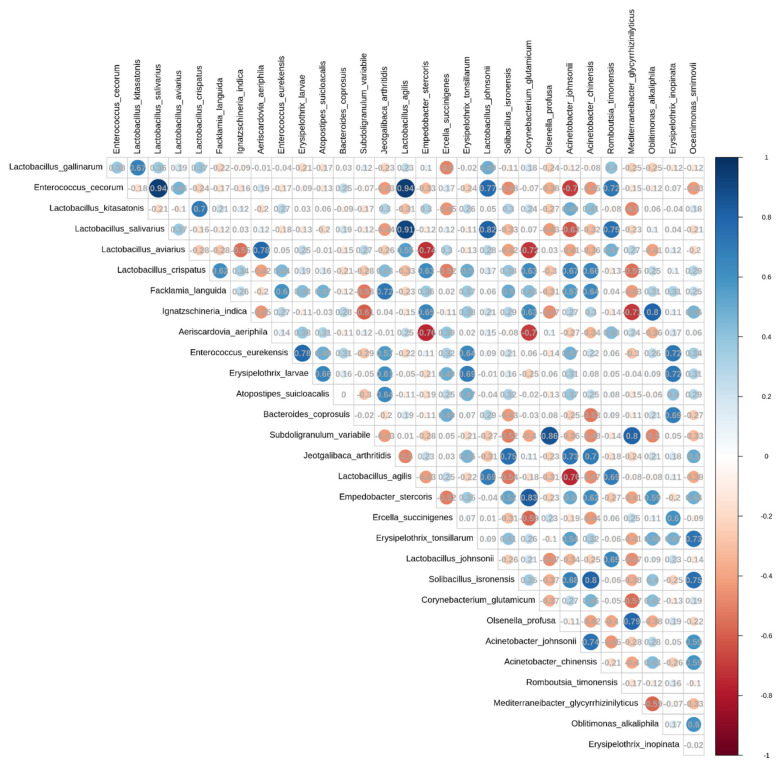
Spearman correlation analysis among the abundance of the top 30 dominant species in the fecal microbiome of the D group. Spearman's rank correlation coefficients were calculated to explore interspecies relationships. The blue indicates positive correlations, and the red signifies negative correlations. The color's intensity corresponds to the correlation's strength, with darker shades indicating stronger positive or negative relationships between species.

Spearman correlation analysis was performed to examine the relationships among significant biomarkers in the D group, using a correlation coefficient threshold 0.7 (Figure 6). The results demonstrated that *Enterococcus cecorum* was positively correlated with *Lactobacillus salivarius*, *Lactobacillus agilis*, and *Romboutsia timonensis*, with the strongest dependency observed for *Lactobacillus salivarius* and *Lactobacillus agilis* (correlation coefficient = 0.94). Besides, *Lactobacillus kitasatonis* showed a positive correlation with *Lactobacillus crispatus*. *Lactobacillus salivarius* exhibited positive correlations with *Lactobacillus agilis*, *Lactobacillus johnsonii*, and *Romboutsia timonensis*, showing the strongest relationship found with *Lactobacillus agilis*, followed by *Lactobacillus johnsonii*. Among non-lactic acid bacteria, *Acinetobacter johnsonii* was positively correlated with *Acinetobacter chinensis*, while *Solibacillus isronensis* displayed positive correlations with both *Acinetobacter chinensis* and *Oceanimonas smirnovii*.

## DISCUSSION

The mEPR was used as a clinical trait to evaluate differences in microbiome composition and diversity between high- and low-productive laying hens. Specific microbial taxa associated with superior performance were further analyzed to identify predictive microbial markers for selecting highly productive hens and to explore potential candidates for enhancing egg production efficiency. Firmicutes and Bacteroidetes, both central to short-chain fatty acid metabolism and energy conversion, have been linked to multiple productivity traits. A higher F/B ratio in gut microbiota has been associated with increased body weight in humans [13] and broilers [14,15]. Similarly, studies utilizing 16S rRNA metagenomics on fecal samples from laying hens revealed that high-productive hens harbor significantly higher relative abundances of Firmicutes compared with their low-productive counterparts [16]. In the present study, the F/B ratio in the high-production group was greater than in the low-production group. Pairwise comparisons of individual farms with the identical breeds (D1-P2, D2-P2, and D3-P2) consistently demonstrated higher F/B ratios in high-performing flocks. A high F/B ratio in cecal microbiota has been associated with more efficient feed energy utilization [17], and Firmicutes remained dominant throughout the laying period in high-producing hens. These findings suggest that F/B ratio may serve as a reliable indicator for evaluating laying performance in hens. It should be noted, however, that no fixed F/B ratio has been established as a universal standard across all laying hen strains and production stages. Most studies have relied on relative comparisons, demonstrating that during peak laying periods, high-producing hens generally exhibit a higher F/B ratio in fecal or intestinal microbiota [16,18]. A higher proportion of Firmicutes relative to Bacteroidetes, which may facilitate intestinal nutrient uptake and energy biosynthesis, thereby contributing to increased egg production [15,19].

In human studies, dietary macronutrients such as proteins, carbohydrates, and lipids have been shown to markedly influence the composition and function of the gut microbiota [20]. In poultry, existing evidence indicates that different sources of medium-chain fatty acids [21] or dietary fibers [14] can alter gut microbial composition, while the impact of rearing environment appears to be more pronounced than that of protein sources. Significant differences in intestinal microbiota have been reported under different housing systems (e.g., caged versus free-range), suggesting that rearing conditions may outweigh dietary protein sources in shaping the microbiome [22]. In the present study, all farms employed the same type of housing system and provided commercial layer feeds. Although different brands of feed were used, these diets were primarily composed of grains, soybean meals, and corn, contained no added medications, and complied with national nutritional composition standards. These measures were intended to minimize potential confounding factors in evaluating the association between the fecal microbiome and laying performance.

Alpha diversity in the fecal microbiome revealed significantly greater species richness in low-productive hens compared with high-productive hens. However, no statistical differences were observed in overall microbial diversity or evenness between the two groups. In contrast, beta diversity analysis using Adonis and PCoA demonstrated clear differences in fecal microbiome composition between high- and low-productive hens. These findings suggest that the observed differences are not driven by overall species diversity but rather by the influence of specific taxa. The PCA further demonstrated five key species contributing to these differences. *Enterococcus cecorum* and *Lactobacillus salivarius* were major contributors distinguishing the two groups, while *Lactobacillus kitasatonis*, *Lactobacillus gallinarum*, and *Aeriscadovia aeriphila* emerged as important members shaping the fecal microbiomes of both high- and low-productive hens. In the mature cecal microbiota of laying hens, the dominant phyla are typically Firmicutes, Bacteroidetes, Proteobacteria, Actinobacteria, and Deferribacteres [23], with Firmicutes, Bacteroidetes, and Proteobacteria consistently forming the core components of the chicken gut microbiota across age groups [24]. Our results are consistent with these observations but further demonstrate notable differences in relative abundances between high- and low-producing hens. Specifically, low-productive hens harbored significantly higher proportions of Bacteroidetes, whereas high-productive hens were characterized by greater abundances of Firmicutes. Heatmap analysis highlighted *Enterococcus* and *Lactobacillus* as the dominant genera within Firmicutes, while *Bacteroides* predominated within Bacteroidetes. Species-level analysis further revealed that high-productive hens exhibited significantly higher relative abundances of *Enterococcus cecorum* and *Lactobacillus kitasatonis*. In contrast, low-productive hens showed elevated levels of *Bacteroides eggerthii* and *Bacteroides coprosuis*. LEfSe analysis supported these findings, identifying the same four species as statistically and biologically relevant marker species for high- and low-productive hens, respectively, underscoring their potential roles in egg production performance. To exclude the breed-related effect on microbiome composition, additional LEfSe comparisons were conducted using fecal samples from high- and low-performing layer farms that exclusively raised Hy-Line hens. These analyses revealed *Enterococcus cecorum*, *Lactobacillus salivarius*, and *Lactobacillus gallinarum* as key biomarkers in the D2-P2 and D3-P2 comparisons, while *Lactobacillus kitasatonis* was identified as a biomarker in the D1-P2 and D3-P2 comparisons. Overall, these results indicate that in addition to *Enterococcus cecorum* and *Lactobacillus kitasatonis*, *Lactobacillus salivarius*, and *Lactobacillus gallinarum* may also play pivotal roles in the gut microbiota of high-productive Hy-Line hens, potentially contributing to enhanced egg production efficiency.

*Enterococcus cecorum*, formerly classified as *Streptococcus cecorum*, is a natural inhabitant of the intestinal tract of vertebrates and represents the most prevalent *Enterococcus* species in laying hens older than 12 weeks [25]. In recent years, outbreaks of *Enterococcus cecorum* infections have been documented in broiler and broiler breeder flocks, frequently associated with arthritis and osteomyelitis [26]. These reports suggest the emergence of novel virulent strains that contribute to increased disease frequency and severity. Pathogenic strains are typically recovered from extraintestinal sites, whereas commensal strains are confined to the gut and display distinct biochemical profiles [27]. The mechanisms underlying the emergence of pathogenic strains remain poorly understood. By contrast, *Enterococcus cecorum* isolated from the intestinal contents of laying hens raised under different housing systems has not been associated with clinical disease or pathogenic traits [28]. Consistent with these findings, the current study demonstrated that *Enterococcus cecorum* was highly abundant in the fecal microbiome of healthy laying hens, supporting its commensal role in this population. Furthermore, correlation analysis revealed that the relative abundance of *Enterococcus cecorum* in high-producing hens was strongly dependent on *Lactobacillus salivarius* and *Lactobacillus agilis*. These findings indicate that *Enterococcus cecorum* may influence the host performance indirectly by modulating the abundance of specific *Lactobacillus* species, thereby contributing to the microbial network associated with enhanced egg production efficiency.

Lactic acid bacteria (LAB), particularly *Lactobacillus* spp., have recently gained recognition as promising probiotic alternatives to in-feed antibiotics in poultry. Their antimicrobial properties and beneficial effects on gut health and immunity are well- established. Previous studies have shown that *Lactobacillus* spp. can enhance growth performance, reduce pathogen colonization, strengthen mucosal integrity, and promote the proliferation of beneficial microbes within the gut microbiome [29]. These effects are largely mediated through the production of metabolites such as bacteriocins, organic acids (e.g., lactate), and hydrogen peroxide, which collectively improve gut environment and pathogen control. Moreover, lactobacilli enhance host immunity and suppress enteric pathogens by reducing gut pH and competing for nutrients and adhesion sites. [30]. As a key LAB genus, *Lactobacillus* plays a pivotal role in optimizing host-microbe interactions. For instance, dietary supplementation with 0.6% metabolites derived from *Lactobacillus plantarum* in diets has been shown to significantly improve egg production in laying hens [31].

*Lactobacillus kitasatonis*, first isolated from the small intestine of chickens, is phylogenetically related to *Lactobacillus amylovorus*, *Lactobacillus crispatus*, and *Lactobacillus acidophilus* [32]. Beyond the small intestine, it has also been identified in the cloaca and feces [33]. Nonetheless, limited studies describe its functional role in poultry health. To our knowledge, this is the first study to report an association between *Lactobacillus kitasatonis* and laying performance in hens. Correlation analysis revealed a positive relationship between *Lactobacillus kitasatonis* and *Lactobacillus crispatus*, alongside a negative relationship with *Bacteroides coprosuis*. Similar to other LAB, *Lactobacillus kitasatonis* is likely to benefit the host by enhancing nutrient absorption and contributing to microbial homeostasis. Notably, *Lactobacillus crispatus* has been reported to inhibit pathogenic bacteria through competitive exclusion and the production of antimicrobial metabolites [34]. Thus, enrichment of *Lactobacillus kitasatonis* in the gut microbiota of laying hens may promote the growth of beneficial microbes such as *Lactobacillus crispatus*, supporting gut health and improving egg productivity.

This study identified *Lactobacillus gallinarum* as a biomarker in the fecal microbiome of high-productive Hy-Line hens, with its abundance showing a positive correlation with *Lactobacillus kitasatonis* (r = 0.67). Previous studies have demonstrated that supplementation with *Lactobacillus gallinarum* reduced cecal colonization by *Campylobacter jejuni* and *Salmonella* in experimentally challenged broilers [35,36]. Moreover, *Lactobacillus gallinarum* has been reported to bind aflatoxin B1 in feed [37] and modulates gut microbiota by enriching beneficial microbes and depleting pathogenic bacteria in mice [38]. Collectively, these attributes suggest that a higher prevalence of *Lactobacillus gallinarum* may provide health benefits to the host. However, its role in egg production efficiency has rarely been investigated in laying hens, and further studies are needed to clarify its physiological effects and potential applications in enhancing production.

*Lactobacillus salivarius* also emerged as a key contributor to the microbial community in laying hens and served as a biomarker in high-performing Hy-Line flocks. This species can ferment a wide range of carbon sources, producing lactic acid as a primary metabolite [39], which underlies its probiotic potential. Supplementation with *Lactobacillus salivarius* and *Lactobacillus agilis* in feed has been shown to increase egg production rates and reduce intestinal *Escherichia coli* populations in Hy-Line hens [40]. Additional studies have demonstrated that dietary *Lactobacillus salivarius* improved growth performance, mitigated organ damage associated with heat stress and *E. coli* infection, and enhanced immune responses in White Leghorn chickens [41]. These benefits indicate that the dominance of *Lactobacillus salivarius* within the gut may support improved performance in laying hens. Although the relative abundance of *Lactobacillus salivarius* did not differ significantly between the D and P groups in this study, LEfSe analysis in high-performing Hy-Line hens underscored its potential role in supporting egg production efficiency, similar to that observed for *Lactobacillus kitasatonis*. Moreover, dietary supplementation with *Lactobacillus salivarius* has also been associated with improvements in egg quality [42]. Taken together, these findings highlight the potential of *Lactobacillus kitasatonis* and *Lactobacillus salivarius*, either alone or in combination, as candidate probiotics to enhance egg production and overall performance in laying hens.

## CONCLUSION

The fecal microbiome of high-performing laying hens was characterized by a greater abundance of *Enterococcus cecorum* and LAB, accompanied by a significantly higher F/B ratio compared with low-performing hens. Among the LAB species, *Lactobacillus kitasatonis*, *Lactobacillus salivarius*, and *Lactobacillus gallinarum* appear to play important roles in the gut microbiota of highly productive hens. While the functional role of *Enterococcus cecorum* remains unclear and requires further investigation, targeted supplementation with the identified *Lactobacillus* species may represent a promising probiotic strategy to enhance egg production efficiency under commercial farming conditions investigated in this study.

## Figures and Tables

**Table 1:** Information on sampled layer farms

Farm type1)	Farm	Number of layers	6-mon2) average egg production rate (amEPR) (%)	Breed	Age (wk)	Fecal samples3)
D	D_1_	9,900	88.26	Hy-Line	58	36
	D_2_	22,000	90.76	Hy-Line	48	36
	D_3_	4,200	84.08	Hy-Line	28	36
P	P_1_	3,600	40.00	HENDRIX	36	36
	P_2_	5,400	53.50	Hy-Line	25	36
	P_3_	18,500	60.12	HENDRIX	38	36

1) D, the group of layer farms with a high egg production rate; P, the group of layer farms with a low egg production rate.

2) Six-month average period spanned from December 2020 to May 2021.

3) Fecal samples were collected randomly from each house on the farms.

**Table 2:** Dissimilarity tests of microbial community composition between D and P groups (Bray Curtis)

Test	Statistic value		p-value
Adonis	F	2.655	0.001
Anosim	R	0.264	0.001
MRPP	δ	0.789	0.001

Adonis, permutational multivariate analysis of variance; Anosim, analysis of similarities; MRPP, multi-response permutation procedure.

## Data Availability

Upon reasonable request, the datasets of this study can be available from the corresponding author.
